# The association between childhood maltreatment and internet addiction among Chinese junior middle school students: prospective cohort study

**DOI:** 10.1192/bjo.2023.606

**Published:** 2023-12-06

**Authors:** Xingyue Jin, Yuxin Wang, Chunxiang Huang, Xuerong Luo, Xueping Gao, Yanmei Shen

**Affiliations:** Department of Psychiatry, National Clinical Research Center for Mental Disorders, and National Center for Mental Disorders, The Second Xiangya Hospital of Central South University, Changsha, Hunan, China

**Keywords:** Maltreatment, abuse, emotional abuse, internet addiction, cohort study

## Abstract

**Background:**

Childhood maltreatment is associated with internet addiction, but most evidence is from retrospective studies.

**Aims:**

We aimed to investigate the relationship between childhood maltreatment and internet addiction through a prospective cohort design.

**Method:**

In a prospective cohort study, self-reported data on childhood maltreatment (Childhood Trauma Questionnaire – Short Form) at baseline, and internet addiction (Revised Chinese Internet Addiction Scale) at baseline and 6-month follow-up, were collected online from 756 Chinese junior middle school students aged 11–15 years and residing in Changsha, Hunan Province. Demographic data and covariates such as depression, anxiety, stress (Depression, Anxiety, and Stress Scale 21) and insomnia (Athens Insomnia Scale) were also surveyed at baseline. Logistic regression analysis measured the association between childhood maltreatment and internet addiction, and gender-related differences.

**Results:**

Childhood maltreatment was prevalent in Chinese junior middle school students (37.83%), and the incidence rate of internet addiction was 9.26% at the 6-month follow-up. Emotional abuse was a significant risk factor for internet addiction (adjusted odds ratio 2.618, 95% CI 1.194–5.738; *P* = 0.016) in both males and females.

**Conclusions:**

This study suggests a high prevalence of childhood maltreatment in Chinese junior middle school students, and that emotional abuse plays a significant role in internet addiction. More attention should be paid to parenting style and adolescents’ mental health.

Internet addiction, sometimes called pathological, problematic or excessive internet use, is characterised by overuse of the internet, a strong desire to re-use the internet and withdrawal reactions when stopping or reducing internet use.^1^ In a previous meta-analysis covering studies from 31 countries, Cheng and Li found that the pooled prevalence rate of internet addiction was about 6.0% in the general population, with the highest prevalence in the Middle East (10.9%) and the lowest in Northern and Western Europe (2.6%).^2^ Among college students in China, a meta-analysis including 70 studies covering 122 454 university students showed that the pooled overall prevalence of internet addiction is 11.4%, with a higher prevalence in boys (13.7%) than girls (6.6%).^3^ The negative impact of internet addiction on physical and mental health has become a global problem requiring further attention. Excessive internet use has been associated with inattention, poor academic performance, headaches, musculoskeletal pain and fatigue,^4^ and psychiatric disorders such as insomnia,^5^ anxiety disorder, depression disorder, attention-deficit hyperactivity disorder, social phobia and obsessive–compulsive disorder.^6^ Considering the effects of internet addiction, identifying potential risk factors associated with internet addiction is essential and could aid in prevention.

Childhood maltreatment refers to adverse experiences that include emotional abuse, physical abuse, sexual abuse, emotional neglect and physical neglect, resulting in actual or potential harm to the child's health, survival, development or dignity in the context of a relationship of responsibility, trust or power.^7^ Not only does it cause instant injury or emotional reactions such as fear, helplessness, sadness and crying, as a long-standing poor parenting style, childhood maltreatment can have many long-term adverse psychological effects on individuals, such as low self-esteem,^8,9^ anxiety,^10^ depression^7,8,10^ and negative cognitions.^9^ Childhood maltreatment exposure is a significant risk factor for mental illnesses such as mood disorders in adulthood.^11^ A clear association was found between childhood maltreatment and mental illness in adulthood, including increased risk, severity and duration of psychotic symptom presentation and worse functional dysfunction.^12^ Previous studies have provided evidence supporting the notion that childhood maltreatment is associated with a higher risk of internet addiction.^8,13,14^ With anonymity, convenience and escape characteristics, the internet provides an ideal platform for self-expression and self-catharsis for individuals with traumatic childhood experiences. The resulting relief of stress and pain in real life may cause increased internet use and therefore increased risk for internet addiction.^15^

## Aims

Although several studies investigating the relationship between childhood maltreatment and internet addiction have been conducted in adolescents^13^ and college students,^14^ most of them adopted a cross-sectional study design, and we were also interested in whether past childhood maltreatment was associated with the emergence of internet addiction. Therefore, further research is needed to explore potential longitudinal associations between these variables. This study aimed to conduct a prospective cohort investigation to evaluate the effect of childhood maltreatment on internet addiction at a 6-month follow-up among Chinese adolescents.

## Method

### Participants

**Fig. 1:**
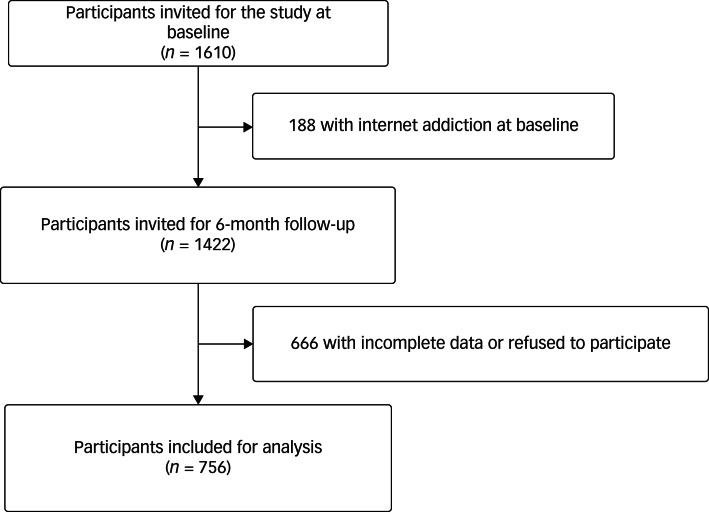
Flow chart of the study progress.

In this prospective cohort study, all students from one junior middle school in seventh, eighth and ninth grade in Changsha, Hunan Province, China, were invited to participate by convenience sampling method. With teachers’ explanations, students were asked to complete online questionnaires at baseline and 6-month follow-up through the WeChat platform. This study collected baseline data from November 2020 to December 2020, and followed up after 6 months. Among the 1610 students invited, 188 with internet addiction were excluded at baseline and 666 were excluded because of incomplete data at 6-month follow-up or refusal to participate in follow-up. A total of 756 students aged between 11 and 15 years were included in the following analysis (Fig. 1). This study was ethically approved by the ethics committee of the Second Xiangya Hospital (approval numbers 2019LSK021 and 2021GLSK013). Every participant and their caregiver signed an online informed consent form. All procedures undertaken in this work adhere to the ethical standards set by the pertinent national and institutional committees on human experimentation, as well as the Helsinki Declaration of 1975, with its 2008 revision.

### Measurement

Demographic characteristics and Childhood Trauma Questionnaire – Short Form (CTQ-SF),^16^ Revised Chinese Internet Addiction Scale (CIAS-R),^17^ Depression, Anxiety, and Stress Scale 21 (DASS-21)^18^ and Athens Insomnia Scale (AIS)^19^ scores were collected at baseline. CIAS-R, DASS-21 and AIS scores were collected at the 6-month follow-up. Demographic characteristics include gender, age, nationality (Han or other) and one-child family (yes or no).

Childhood maltreatment was evaluated with the CTQ-SF at baseline.^16^ The CTQ-SF is a self-reported scale with 28 items assessing five categories of childhood maltreatment: physical abuse, emotional abuse, physical neglect, emotional neglect and sexual abuse. Each item was measured on a five-point Likert response from ‘never happened’ (score 0) to ‘always happened’ (score 4). The Chinese version of the CTQ-SF also has good reliability and validity.^16^ We measured four types of childhood maltreatment (physical abuse, emotional abuse, physical neglect and emotional neglect) ever experienced by adolescent participants. The cut-off scores were as follows: ≥10 for physical abuse, ≥13 for emotional abuse, ≥10 for physical neglect and ≥15 for emotional neglect.^20^ Scores equal to higher than the cut-off scores were considered to constitute maltreatment. The childhood maltreatment group comprised participants who had experienced one or more kinds of childhood maltreatment.

Internet addiction was evaluated by the CIAS-R.^17^ CIAS-R is a 19-item self-report scale designed to measure internet use. Each item was measured on a four-point scale from ‘complete inconformity’ (score 0) to ‘complete conformity’ (score 3). Participants with a total score of ≥53 comprised the internet addiction group.^17^ The CIAS-R has good reliability and validity in the Chinese population.^17^

Three subscales of the DASS-21 were used to assess stress, depression and anxiety at baseline over the past week.^18^ Each item was measured on a four-point Likert scale from ‘complete inconformity’ (score 0) to ‘complete conformity’ (score 3). The higher the total scores of subscales, the more severe the symptoms. The cut-off scores for each subscale were ≥11 for depression, ≥8 for anxiety and ≥13 for stress; participants scoring equal to or higher than the cut-off were considered as having severe symptoms, and we classified them as having depression, anxiety or stress, respectively. The Chinese short version of DASS-21 has good reliability and validity.^18^

The AIS was used to measure insomnia symptoms. Participants with a total score higher than six were considered to have clinically significant insomnia.^19^ The AIS has good psychometric properties in the Chinese population.^19^

### Statistical analysis

The *χ*^2^-test was employed to compare group differences in participants with and without childhood maltreatment during categorical variables such as gender, nationality, one-child family, stress, anxiety, depression, insomnia and internet addiction. The Mann–Whitney *U*-test was employed to compare group differences in age. Logistic regression was utilised to analyse the association between childhood maltreatment and internet addiction, and any gender-related differences. The exposure of childhood maltreatment and each specific type of childhood maltreatment were included in logistic regression analysis to analyse their relationships with internet addiction in the total sample and stratified by gender. The *P-*value for interaction between gender and childhood maltreatment or its subtypes in logistic regression was utilised to compare gender-related differences. Overall, model 1 was the crude model and no confounders were controlled; potential confounders were adjusted in model 2 (adjusted for age, gender, nationality, one-child family, anxiety, depression and stress) and model 3 (adjusted for age, gender, nationality, one-child family, anxiety, depression, stress and insomnia). The odds ratio was used to show statistical associations and the effect size of childhood maltreatment.^21^ A *P* < 0.05 (two-tailed) was considered statistically significant. All analyses were conducted with SPSS version 25.0 for Windows (IBM Corporation, Illinois, USA).

## Results

Among the 756 students who participated in the survey at baseline and 6-month follow-up, the incidence rate of internet addiction was 9.26% (70/756) at the 6-month follow-up. A total of 286 (37.83%) students had experienced childhood maltreatment at baseline. Specifically, 73 (9.66%) students had experienced emotional abuse, 58 (7.67%) had experienced physical abuse, 177 (23.41%) had experienced emotional neglect and 194 (25.66%) had experienced physical neglect. A total of 139 (18.39%) students had experienced one type of childhood maltreatment and 147 (19.44%) students had experienced more than one kind of childhood maltreatment. Students who experienced childhood maltreatment were more likely to be from one-child families (*P* = 0.017) and have symptoms of stress, anxiety, depression or insomnia (*P* < 0.001) (Table 1). Additionally, for the 188 students with internet addiction at baseline that we excluded, 96 (51.06%) were lost to follow-up, 32 (17.02%) had lower CIAS-R scores than the cut-off and 60 (31.91%) still had an internet addiction at 6-month follow-up.

**Table 1:** Group differences between participants with and without childhood maltreatment

	Participants without childhood maltreatment *N* = 470		Participants with childhood maltreatment *N* = 286		*Z*/*χ*^2^	*P-*value
	*n* (mean)	% (s.d.)	*n* (mean)	% (s.d.)		
Age, years, mean (s.d.), *Z*	12.59	0.62	12.62	0.65	−0.732	0.464
Gender, *χ*2						
Male, *n* (%)	250	53.2	150	52.4	0.039	0.842
Female, *n* (%)	220	46.8	136	47.6		
Nationality, *χ*2						
Han, *n* (%)	450	95.7	272	95.1	0.169	0.681
Other, *n* (%)	20	4.3	14	4.9		
One-child family, *χ*2						
No, *n* (%)	209	44.5	102	35.7	5.691	0.017*
Yes, *n* (%)	261	55.5	184	64.3		
Stress, *χ*2						
No, *n* (%)	399	84.9	195	68.2	29.494	<0.001***
Yes, *n* (%)	71	15.1	91	31.8		
Anxiety, *χ*2						
No, *n* (%)	276	58.7	119	41.6	20.875	<0.001***
Yes, *n* (%)	194	41.3	167	58.4		
Depression, *χ*2						
No, *n* (%)	362	77.0	155	54.2	42.849	<0.001***
Yes, *n* (%)	108	23.0	131	45.8		
Insomnia, *χ*2						
No, *n* (%)	284	60.4	103	36.0	42.407	<0.001***
Yes, *n* (%)	186	39.6	183	64.0		

*P < 0.05, ***P < 0.001.

In the logistic regression, childhood maltreatment had no significant influence on the incident of internet addiction in both model 1 (crude odds ratio 0.787, 95% CI 0.467–1.326; *P* = 0.369), model 2 (adjusted for age, gender, nationality, one-child family, anxiety, stress and depression at baseline; adjusted odds ratio 0.738, 95% CI 0.428–1.274; *P* = 0.276) and model 3 (adjusted for age, gender, nationality, one-child family, anxiety, stress, depression and insomnia at baseline; adjusted odds ratio 0.720, 95% CI 0.415–1.246; *P* = 0.240). However, when considering concrete types of childhood maltreatment, emotional abuse was significantly associated with internet addiction in both model 1 (crude odds ratio 2.576, 95% CI 1.300–5.105; *P* = 0.007), model 2 (adjusted odds ratio 2.667, 95% CI 1.224–5.809; *P* = 0.014) and model 3 (adjusted odds ratio 2.618, 95% CI 1.194–5.738; *P* = 0.016). Physical abuse, emotional neglect and physical neglect showed no significant effect on internet addiction (*P* > 0.05) (Table 2).

**Table 2:** The association of internet addiction and childhood maltreatment, by logistic regression

	Model 1			Model 2			Model 3		
	Odds ratio	95% CI	*P-*value	Odds ratio	95% CI	*P-*value	Odds ratio	95% CI	*P-*value
Childhood maltreatment	0.787	0.467–1.326	0.369	0.738	0.428–1.274	0.276	0.720	0.415–1.246	0.240
Emotional abuse	2.576	1.300–5.105	0.007**	2.667	1.224–5.809	0.014*	2.618	1.194–5.738	0.016*
Physical abuse	1.641	0.744–3.618	0.219	1.440	0.619–3.349	0.397	1.420	0.610–3.304	0.416
Emotional neglect	0.653	0.342–1.246	0.196	0.616	0.317–1.196	0.152	0.598	0.306–1.165	0.131
Physical neglect	0.846	0.472–1.515	0.573	0.805	0.444–1.459	0.474	0.797	0.439–1.445	0.454

Model 1: crude odds ratio. Model 2: adjusted for age, gender, nationality, one-child family, anxiety, depression and stress. Model 3: adjusted for age, gender, nationality, one-child family, anxiety, depression, stress and insomnia.*P < 0.05, **P < 0.01.

Considering the possible gender-related differences, we applied logistic regression on males and females separately, and explored its interaction. There was no significant gender difference in the influence of childhood maltreatment and its subtypes and internet addiction (*P*-value for interaction > 0.05). However, unexpectedly, emotional neglect was negatively associated with internet addiction in males in model 3 (adjusted odds ratio 0.359, 95% CI 0.135–0.959; *P* = 0.041), but the *P*-value for interaction was not significant in males and females (*P*-value for interaction = 0.115) (Table 3).

**Table 3:** The gender difference in the association between internet addiction and childhood maltreatment, by logistic regression

		Model 1			Model 2				Model 3			
	Gender	Odds ratio	95% CI	*P-*value	Odds ratio	95% CI	*P-*value	*P*-value for interaction	Odds ratio	95% CI	*P-*value	*P*-value for interaction
Childhood maltreatment	Male	0.670	0.339–1.326	0.250	0.646	0.317–1.316	0.228	0.520	0.615	0.301–1.257	0.183	0.548
	Female	1.012	0.445–2.299	0.977	0.868	0.365–2.066	0.749		0.960	0.394–2.338	0.928	
Emotional abuse	Male	3.018	1.202–7.576	0.019*	3.554	1.190–10.618	0.023*	0.690	3.397	1.132–10.134	0.029*	0.675
	Female	2.381	0.837–6.770	0.104	2.025	0.628–6.522	0.237		2.567	0.735–8.967	0.140	
Physical abuse	Male	1.545	0.608–3.927	0.360	1.566	0.564–4.349	0.390	0.948	1.522	0.547–4.240	0.421	0.933
	Female	1.534	0.335–7.035	0.582	1.180	0.241–5.767	0.838		1.331	0.267–6.636	0.727	
Emotional neglect	Male	0.390	0.149–1.021	0.055	0.382	0.144–1.019	0.055	0.110	0.359	0.135–0.959	0.041*	0.115
	Female	1.211	0.497–2.987	0.678	1.079	0.415–2.804	0.876		1.181	0.446–3.129	0.738	
Physical neglect	Male	0.561	0.252–1.249	0.157	0.541	0.239–1.226	0.141	0.119	0.532	0.234–1.209	0.132	0.125
	Female	1.460	0.611–3.489	0.394	1.353	0.556–3.290	0.505		1.431	0.582–3.519	0.436	

Model 1: crude odds ratio. Model 2: adjusted for age, gender, nationality, one-child family, anxiety, depression and stress. Model 3: adjusted for age, gender, nationality, one-child family, anxiety, depression, stress and insomnia.*P < 0.05.

## Discussion

The present study explored the association between childhood maltreatment and internet addiction, and its gender-related differences, by prospective cohort design. This study found a high prevalence of childhood maltreatment in Chinese junior middle school students (37.83%). Emotional abuse was a significant risk factor for internet addiction in males and females.

The results showed that a high percentage of junior middle school students (37.83%) had experienced childhood maltreatment. A systematic review found that 64.7% (95% CI 52.3–75.6%) of Chinese college students had experienced childhood maltreatment.^22^ Because of the young age of the population we investigated, the prevalence of childhood maltreatment was lower than in some previous studies, but it still showed a high prevalence of childhood maltreatment; nearly 20% of students had experienced more than one subtype of childhood maltreatment. Childhood maltreatment is a universal phenomenon that needs more attention. Consistent with previous studies,^7,11,12^ we also found that students who had experienced childhood maltreatment were more likely to experience stress, anxiety, depression and insomnia. The hopelessness theory of depression is a prominent cognitive model of depression, which states that childhood maltreatment, especially emotional abuse, results in negative inferential styles, and these styles contribute to the path from adverse life events to hopelessness, and then on to hopelessness, depression, and suicidal ideation and behaviours.^23^ Moreover, individuals who had experienced childhood maltreatment were more likely to have impaired emotional regulation and develop poor emotional regulation patterns,^24^ harming their mental and psychological health. In addition, students with internet addiction had a high prevalence of insomnia,^25^ which was found to be an independent risk factor for internet addiction.^26^ Similarly, a path analysis among Chinese college students suggested a significant mediator role of sleep duration between emotional abuse and internet addiction,^14^ so we considered insomnia as a confounding factor between childhood maltreatment and internet addiction, and adjusted together with depression, stress and anxiety in the models.

After controlling for these confounding factors, we also found that emotional abuse at baseline was associated with internet addiction at 6-month follow-up, consistent with previous studies,^27^ and highlighted the importance of emotional abuse. Emotional abuse refers to the long-term use of incorrect parenting styles by parents or caregivers, such as blame, ridicule, rejection and hostility.^28^ For emotion dysregulation we mentioned above was also independently associated with emotional abuse^29^ and was a predictor for all addictive behaviours, including internet addiction.^30^ The pathway analysis showed that emotion dysregulation mediated the relationship between emotional abuse and internet gaming addiction, a subtype of internet addiction.^31^ Moreover, individuals with childhood maltreatment often reported lower family support,^32^ and individuals with emotional abuse may receive insufficient family emotional support. Adolescents with internet addiction perceived their parents as emotionally distant, poor or absent in caregiving or supervising.^33^ Estévez et al thought adolescents might use the internet excessively because of insufficient attachment to their parents.^30^ There was a vicious circle of childhood maltreatment, internet addiction and lower family support. Individuals experiencing childhood maltreatment often received lower family support,^32^ and individuals with childhood maltreatment provided less emotional support to their family members, including children,^34^ which was another form of emotional abuse and increased the risk of internet addiction. Because of the high prevalence of internet addiction reported in males,^3^ we have taken gender differences into account and found no significant differences in the relationship between emotional abuse and internet addiction between male and female students. In model 2, emotional neglect was even a protective factor for internet addiction in males; these relationships in males and females may differ from the whole sample because of the different subsamples, and gender-related effects should be explored in a larger population.

However, in this study, we did not find associations between general childhood maltreatment and other subtypes of childhood maltreatment (emotional neglect, physical abuse and physical neglect) with internet addiction. Previous cross-sectional studies showed direct associations between childhood maltreatment and internet addiction in adolescents,^13^ and college students with internet addiction exhibited a higher level of emotional and physical abuse.^14^ In one cohort study, Geng et al found that childhood maltreatment and conflicts between parents predicted longitudinal moderate or higher levels of problematic internet use in adolescents.^35^ Some research has partly explained the mechanism: maladaptive cognitive emotion regulation strategies,^13^ depression,^13,36^ family support,^37^ bullying perpetration^37^ and bullying victimisation^37^ were considered essential mediators relating to childhood maltreatment and internet addiction. For adolescents with more severe childhood maltreatment (total CTQ-SF ≥42), depression plays a complete mediating role in the relationship between childhood maltreatment and internet addiction.^36^ According to the biopsychosocial model of addiction^38^ and social compensation hypothesis,^39^ childhood maltreatment is an essential social and environmental factor representing a long-standing unhealthy parenting style, causing an unsafe environment. Individuals in such environments would prefer not to disclose themselves in real life and would prefer to form close relationships on the internet, which increases the risk of internet addiction. Although our study did not find associations between childhood maltreatment, physical abuse, physical neglect and emotional neglect and internet addiction, these relationships are potential and need to be explored in a larger sample with a longer follow-up period.

The present study has several limitations. First, as the participants we recruited were all from one junior middle school in Hunan Province, with a relatively small sample size and high rate of loss to follow-up, our results cannot be generalised to the general adolescent population in China. Future studies with larger sample sizes and employing more representative and diverse samples are needed. Second, all scales used in this study were self-rated, which might be subjective and result in reporting bias. A combination of multiple report approaches (such as interviews, observations and objective behavioural measures) and investigation of more potential confounding factors could be used to increase the validity of future results. Third, sexual abuse is an essential form of childhood maltreatment; although we did not investigate it in this study, future studies should make efforts to explore it. Also, future studies should investigate more details, such as specific subtypes of internet addiction. Moreover, although this study used a longitudinal research design, the follow-up period was 6 months; future studies with long-term follow-up periods are needed to better explore the association between childhood maltreatment and internet addiction.

In conclusion, the present study showed that childhood maltreatment was widespread among Chinese junior middle school students; one out of three students reported experiencing childhood maltreatment. Although these results did not establish the relationship between childhood maltreatment and internet addiction, we found that emotional abuse was significantly associated with the occurrence of internet addiction at 6-month follow-up in both males and females. This study highlighted the importance of preventing childhood maltreatment, providing psychoeducation for parents and caregivers on how to take good care of children and adolescents, and caring for adolescents to help them overcome the consequences of childhood maltreatment.

## Data Availability

The supporting data for this study are accessible upon request from the corresponding author, Y.S. However, please note that the data cannot be made publicly available due to privacy or ethical restrictions.
